# Supplementary material for: Near-room temperature ferromagnetic insulating state in highly distorted LaCoO2.5 with CoO5 square pyramids
Source: Nat Commun. 2021 Mar 25;12:1853. doi: 10.1038/s41467-021-22099-y (PMC7994406; doi:10.1038/s41467-021-22099-y)
Supplement: Supplementary file 1 — Supplementary Information [file 41467_2021_22099_MOESM1_ESM.pdf]

Supplementary information for

Near-Room Temperature Ferromagnetic Insulating State in Highly Distorted $LaCoO_{2.5}$ with CoO_5 Square Pyramids

Qinghua Zhang[1,2], Ang Gao[1,3], Fanqi Meng[1, 3], Qiao Jin[1,3], Shan Lin[1,3], Xuefeng Wang[1], Dongdong Xiao[1], Can Wang[1,3,4], Kui-juan Jin[1,3,4], Dong Su[1], Er-Jia Guo[1,4, 5 *], and Lin Gu [1, 3, 4, *]

[1]*Beijing National Laboratory for Condensed Matter Physics, Institute of Physics, Chinese Academy of Sciences, Beijing 100190, China*

[2]*Yangtze River Delta Physics Research Center Co. Ltd., Liyang 213300, China*

[3]*School of Physical Sciences, University of Chinese Academy of Sciences, Beijing 100049, China*

[4]*Songshan Lake Materials Laboratory, Dongguan, Guangdong 523808, China*

[5]*Center of Materials Science and Optoelectronics Engineering, University of Chinese Academy of Sciences, Beijing 100049, China*

*E-mail: ejguo@iphy.ac.cn; l.gu@iphy.ac.cn

I. Microstructural characterizations and EELS analysis

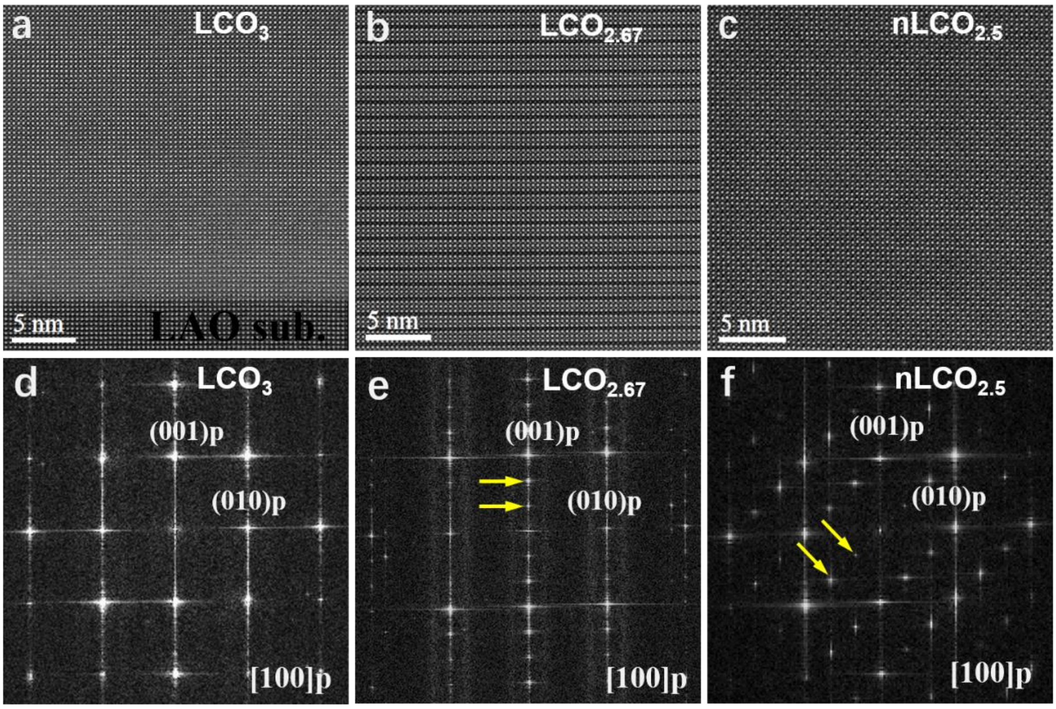

Supplementary Fig. 1. High-magnification high-angle angular dark-field (HAADF) images of (a) pristine LCO_3, (b) $LCO_{2.67}$ and (c) $nLCO_{2.5}$ films grown on LAO substrates. The representative HAADF-STEM images were chosen randomly. For a LCO_3, no vertical or horizontal dark stripes were observed. For a $LCO_{2.67}$, we observed the horizontal dark stripes with a period of $3a_0$. For a $nLCO_{2.5}$, we observed more dense dark stripes aligned 45 degrees to the surface and interface. Such structural modulation is uniform within the entire $nLCO_{2.5}$ sample. The corresponding FFT patterns of (d) pristine LCO_3, (e) $LCO_{2.67}$ and (f) $nLCO_{2.5}$ films are in good agreement with the SAED patterns shown in Figures 1a-c.

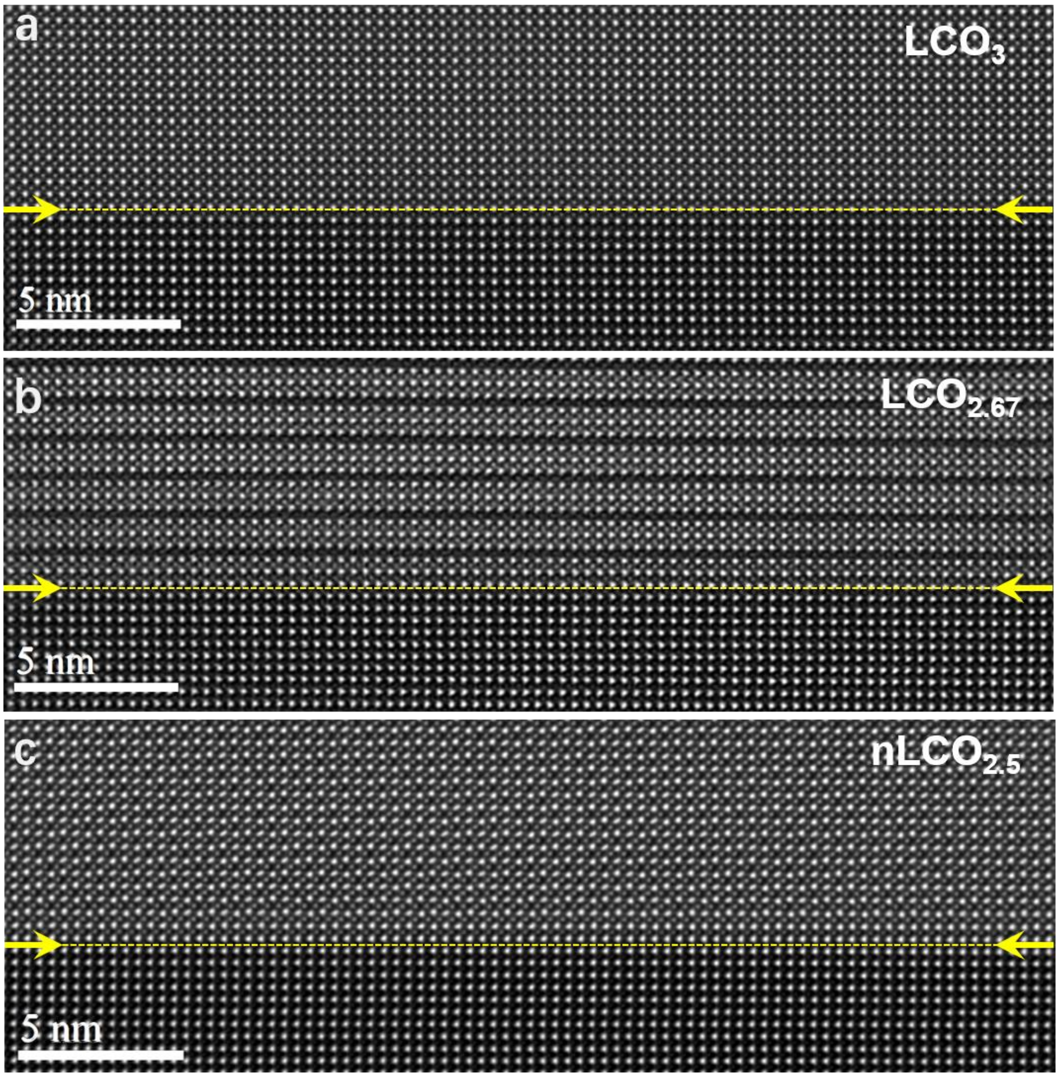

Supplementary Fig. 2. HAADF images of the interfaces between $LaAlO_3$ substrate and LCO_3 (a), $LCO_{2.67}$ (b) and $nLCO_{2.5}$ (c) films, respectively. Yellow arrows and dotted lines indicate the positions of the atomically sharp interfaces and highly coherent epitaxy of films.

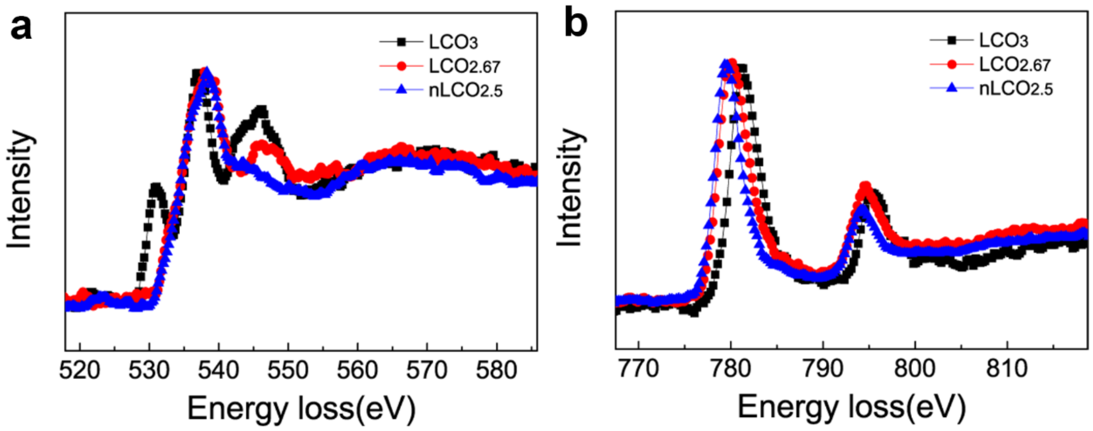

Supplementary Fig. 3. Comparation on the O *K*-edges (a) and Co *L*-edges (b) in three electronic states of LCO_3, $LCO_{2.67}$, and $nLCO_{2.5}$ thin films.

II. Macroscopic structural and electronic state property characterizations

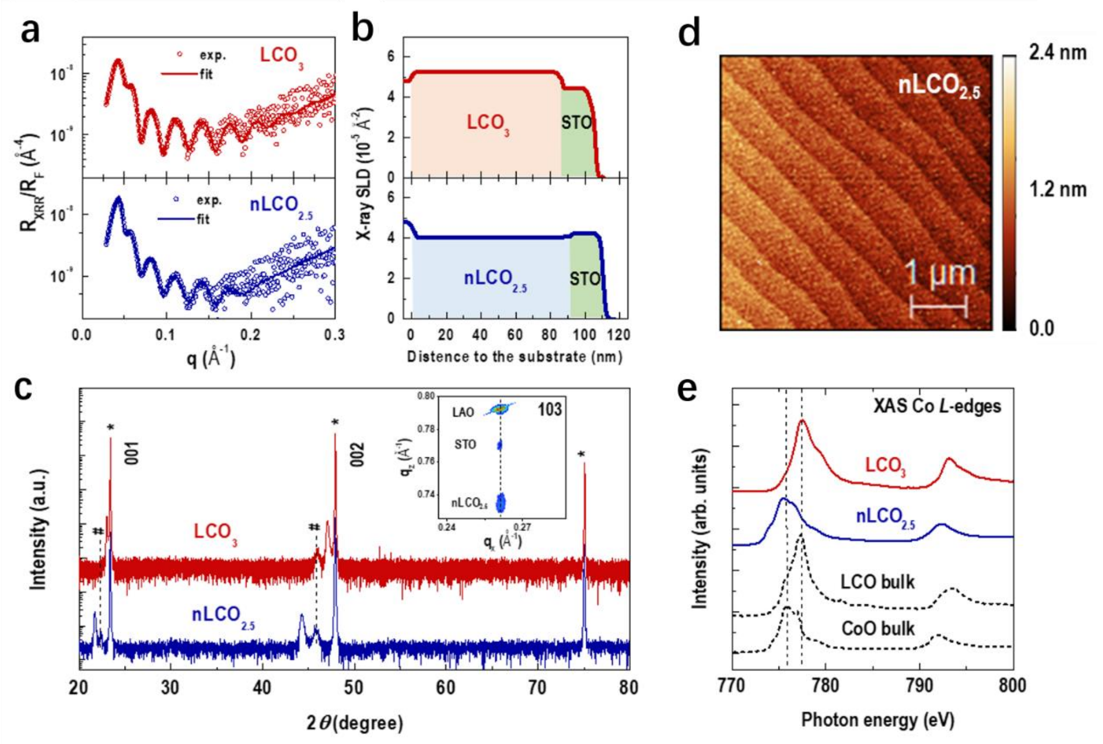

Supplementary Fig. 4. Structure, morphology, and electronic state characterizations in LCO_3 and $nLCO_{2.5}$ thin films. (a) X-ray reflectivities of LCO_3 and $nLCO_{2.5}$ films. The open circular symbols represent the experimental data, and the solid lines are the best fit. From XRR fittings, the chemical depth profiles [shown in (b)] of those two films can be obtained. XRR results indicate the bilayers have uniform chemical distributions, sharp interfaces, and smooth surfaces. The x-ray scattering length density (SLD), which is proportional to its chemical density, of LCO_3 is ~24% larger than that of $nLCO_{2.5}$. In addition, the thicknesses of LCO and STO layers in the STO/LCO_3 bilayer are approximately 84 nm and 20 nm, respectively. While, the thickness of $nLCO_{2.5}$ is ~ 7 nm larger than that of LCO_3, whereas the thickness of STO is unchanged. (c) X-ray diffraction θ-2θ scans of LCO_3 and $nLCO_{2.5}$ films. * indicates the 00*l* peak positions of LAO substrate. # indicates the 00*l* peak positions of STO capping layer. Both curves reveal that the LCO_3 and $nLCO_{2.5}$ films are single-phase and of high structural quality. The out-of-plane lattice constant of LCO_3 is 3.854 Å. After annealing in vacuum for 4 hours, the out-of-plane lattice constant of $nLCO_{2.5}$ films increases to 4.088 Å. The elongation of lattice constant reaches ~ 6.1%, in consistent

with the increment of $nLCO_{2.5}$ layer's thickness. In contrast, the lattice constant of STO layer keeps almost constant, indicating the vacuum-annealing, at least under the same conditions, does not create significant Vo in STO. We also performed reciprocal space mapping (RSM) on a $nLCO_{2.5}$ sample. Inset of (c) shows the RSM around 103 reflections around LAO substrate. Both $nLCO_{2.5}$ layer and STO capping layer are coherently grown on LAO substrate, suggesting the identical in-plane lattice constant. The in-plane strain can be maintained up to ~ 200 u.c. due to the rather small lattice mismatch of ~ 1% between LAO and LCO. (d) Topography of a $nLCO_{2.5}$ film capped with STO layer. Atomic force microscopy (AFM) image was recorded over an area of 5×5 μm^2 using Asylum Research AFM operated in the tapping mode. Surprisingly, we observe the step-and-terrace feature in our sample even without chemical etching the LAO substrate. The averaged r.m.s. is ~ 0.4 nm, revealing a smooth surface. The macroscopic structural and morphology measurements indicate that the as-grown LCO_3 films and newly formed $nLCO_{2.5}$ films are chemically uniform, highly crystalline, and single-phase. (e) X-ray absorption spectra (XAS) for LCO_3 and $nLCO_{2.5}$ films at Co *L*-edges. The reference spectra for Co^{3+} (from bulk LCO) [E. J. Guo *et al*. Science Advances 5, eaav5050 (2019)] and Co^{2+} (from bulk CoO) [N. Lu *et al*. Nature 546, 124 (2017)] are shown as black dashed lines for comparison. Apparently, XAS signal from LCO_3 at Co *L*-edges is nearly dominated by Co^{3+}. The main peak left-shifts to ~ 777 eV in the XAS spectra from $nLCO_{2.5}$. The line shape of XAS from $nLCO_{2.5}$ is almost identical to that of bulk CoO, suggesting that the Co^{3+} ions in LCO_3 transit into Co^{2+} ions in $nLCO_{2.5}$.

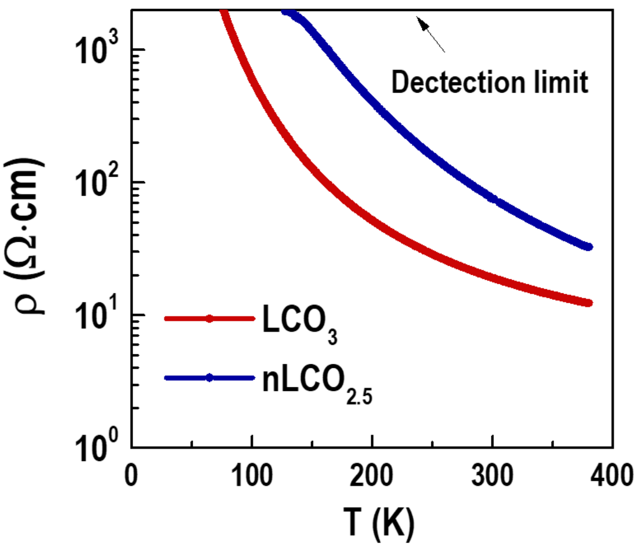

Supplementary Fig. 5. Transport measurements on LCO_3 and $nLCO_{2.5}$ thin films. Resistivity (ρ) of LCO_3 and $nLCO_{2.5}$ thin films were measured using standard van der Pauw method. The room-temperature resistivities of LCO_3 and $nLCO_{2.5}$ thin films are above 1 Megaohms, corresponding to ~ 1 Ω·cm. The *ρ*-*T* curves for LCO_3 and $nLCO_{2.5}$ thin films were measured up to 380 K. The low temperature resistivities were recorded until the detection limit of our electrical device. The negative slopes of *ρ*-*T* curves indicate the insulating character of both LCO_3 and $nLCO_{2.5}$ films.

III. Magnetic properties of $LCO_{2.67}$ films

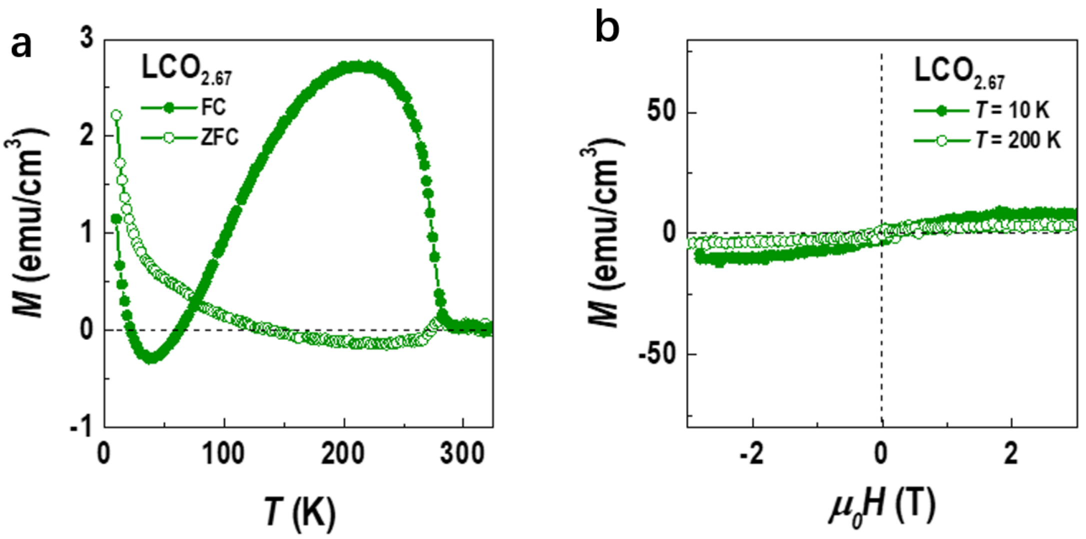

Supplementary Fig. 6. Magnetic properties of $LCO_{2.67}$ films. (a) *M-T* curves measured during the sample warming up at a magnetic field of 0.1 T. Solid and open circles represent the magnetizations were recorded after field-cooled (FC) and zero-field-cooled (ZFC), respectively. (b) *M-H* curves recorded at 10 K and 200 K. The saturation magnetization at 10 K and 200 K is ~ 0.067 μ_B/Co and ~ 0.02 μ_B/Co, respectively.

IV. Spectrum images of O *K*-and Co *L*-edges for the line scan EELS data

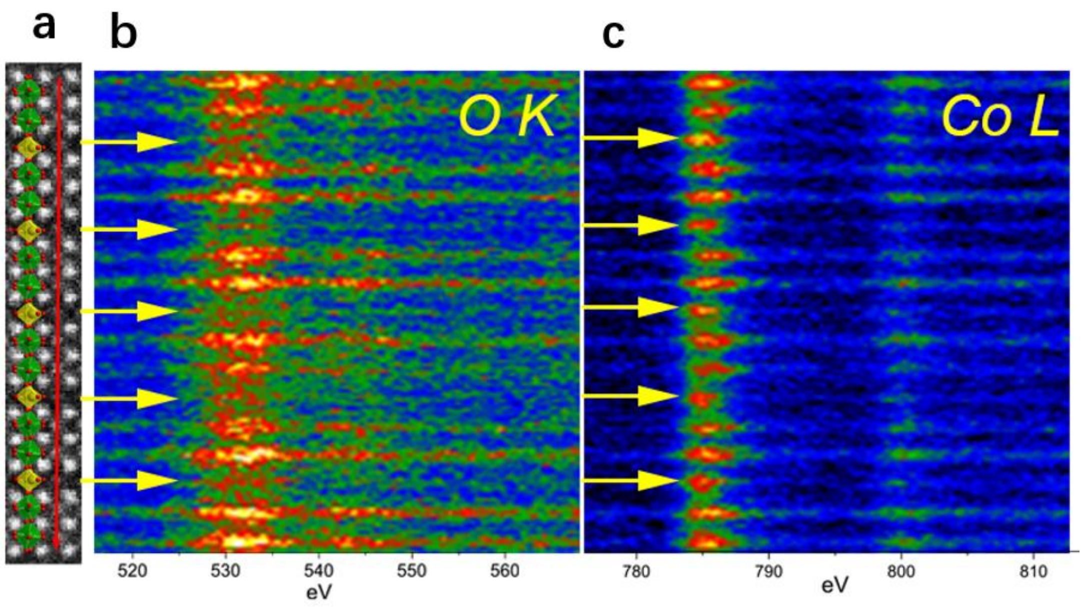

Supplementary Fig. 7. Atomic-resolved EELS spectra of the $LCO_{2.67}$ phase. (a) HAADF image with overlaid CoO_6 octahedra and CoO_4 tetrahedra. The red line indicates the position of line scanning. Two-dimensional spectra image of (b) O *K*- and (c) Co *L*-edges, the yellow arrows indicate the oxygen-deficient CoO_4 tetrahedra. The difference of O *K*- and (c) Co *L*-edges between CoO_6 octahedra and CoO_4 tetrahedra can be identified clearly, with periodicity of $3a_0$.

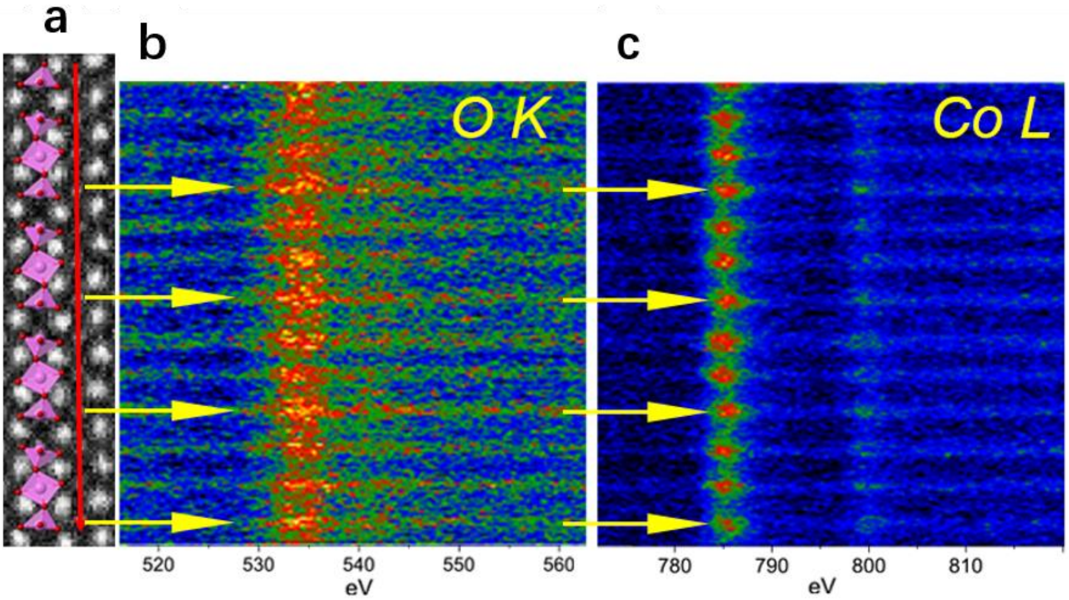

Supplementary Fig. 8. Atomic-resolved EELS spectra of the nLCO$_{2.5}$ **phase.** (a) HAADF image with overlaid CoO$_5$ square pyramids. The red line indicates the position of line scanning. Two-dimensional spectra image of (b) O *K*- and (c) Co *L*-edges, the yellow arrows indicate the - Co columns in Co$_2$-O$_5$ pyramids. A roughly uniform intensity distribution of O *K*- and Co *L*-edges' signals can be visualized.

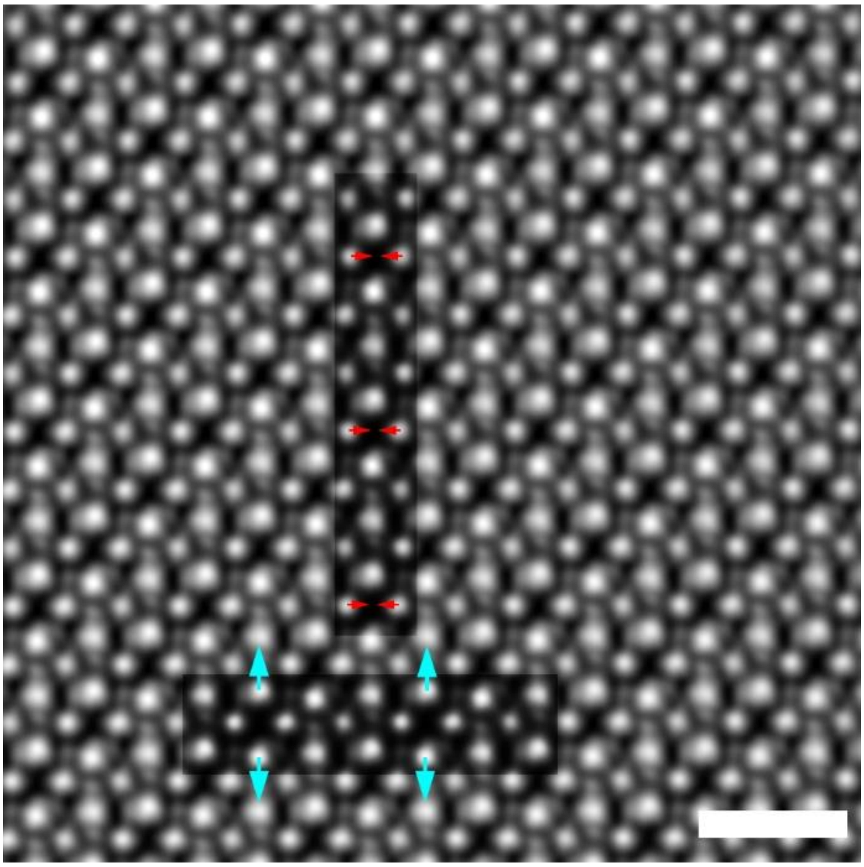

Supplementary Fig. 9. The iDPC image of $nLCO_{2.5}$ **phase.** Image contrast in two rectangular regions were adjusted to highlight atomic displacements of La and Co, as indicated by blue and red arrows, respectively. The scale bar is 1 nm.

V. Structure determination and the summaries of structure parameters

With the accurate projected position of each atom, we construct two most possible structural models (nLCO-A and nLCO-B) as shown in Supplementary Fig. 9. The difference between them is relative Vo position of the Co_2-O_5 square pyramids in the projected direction, where Vos reside alternatively in $(100)_p$ plane among neighbored Co_2-O_5 square pyramids in nLCO-A and in the same $(100)_p$ plane in nLCO-B. We optimized these two structures and find the energy of the nLCO-A is lower than that of nLCO-B, which is also favorable for the alternative arrangement of Vo in the neighbored Co_2-O_5 square pyramids.

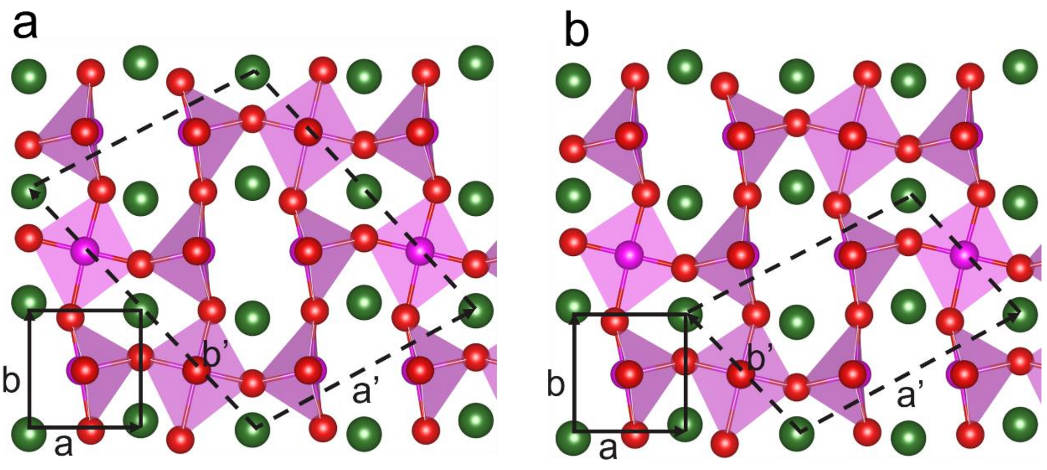

Supplementary Fig. 10. Two possible crystal structures of $nLCO_{2.5}$**.** ***a***, ***b*** are the basic vectors of the unit cell (1×1×1) and ***a'***, ***b'*** are the basic vectors of the extended cell (√5×2√2×2) for (a) nLCO-A and (√5×√2×2) for (b) nLCO-B, respectively, as indicated by dark dotted lines. The corresponding transformation matrix with the basic unit cell is [2, -1, 0; 2, 2, 0; 0, 0, 2] and [2, -1, 0; 1, 1, 0; 0, 0, 2], respectively.

Supplementary Table 1.

Structure parameters summary of the $nLCO_{2.5}$ phase from DFT calculation

supercell	***a*** (Å)	***b*** (Å)	***c*** (Å)	α (°)	β (°)	γ (°)
√5×2√2×2	8.589	11.079	7.58	90.00	90.00	105.11
1×1×1	3.79	4.04	3.79	90.00	90.00	90.00

Atoms	label	number	*x*	*y*	*z*
La	La1	1	-0.007	0.015	0.000
La	La2	1	0.017	0.478	0.000
La	La3	1	0.017	-0.022	0.500
La	La4	1	0.993	0.515	0.500
La	La5	1	0.687	0.193	0.000
La	La6	1	0.687	0.702	0.000
La	La7	1	0.687	0.202	0.500
La	La8	1	0.687	0.693	0.500
La	La9	1	0.323	0.317	0.000
La	La10	1	0.322	0.813	0.000
La	La11	1	0.322	0.313	0.500
La	La12	1	0.323	0.817	0.500
Co	Co1	1	0.344	0.084	0.249
Co	Co1	1	0.344	0.584	0.251
Co	Co1	1	0.344	0.084	0.751
Co	Co1	1	0.344	0.584	0.749
Co	Co1’	1	0.658	0.427	0.247

Co	Co1’	1	0.658	0.927	0.253
Co	Co1’	1	0.658	0.427	0.753
Co	Co1’	1	0.658	0.927	0.747
Co	Co2	1	0.004	0.247	0.273
Co	Co2	1	0.004	0.747	0.227
Co	Co2	1	0.004	0.247	0.727
Co	Co2	1	0.004	0.747	0.773
O	O1	1	0.342	0.076	0.000
O	O2	1	0.342	0.576	0.000
O	O3	1	0.342	0.076	0.500
O	O4	1	0.342	0.576	0.500
O	O5	1	1.000	0.250	0.000
O	O6	1	1.000	0.750	0.500
O	O7	1	0.655	0.418	0.000
O	O8	1	0.655	0.918	0.000
O	O9	1	0.655	0.418	0.500
O	O10	1	0.655	0.918	0.500
O	O11	1	0.131	0.128	0.279
O	O12	1	0.130	0.628	0.221
O	O13	1	0.131	0.128	0.721
O	O14	1	0.130	0.628	0.779
O	O15	1	0.865	0.365	0.276

O	O16	1	0.865	0.865	0.224
O	O17	1	0.865	0.365	0.724
O	O18	1	0.865	0.865	0.776
O	O19	1	0.232	0.416	0.250
O	O20	1	0.232	0.916	0.250
O	O21	1	0.232	0.416	0.750
O	O22	1	0.232	0.916	0.750
O	O23	1	0.777	0.101	0.251
O	O24	1	0.777	0.601	0.249
O	O25	1	0.777	0.101	0.749
O	O26	1	0.777	0.601	0.751
O	O27	1	0.476	0.255	0.250
O	O28	1	0.476	0.755	0.250
O	O29	1	0.476	0.255	0.750
O	O30	1	0.476	0.755	0.750

Supplementary Table 2

The Co-O bond length of CoO_5 square pyramids in $nLCO_{2.5}$ phase from DFT calculation

bond length (Å)	O1	O2	O3	O4	O5
Co1	2.027	1.908	1.937	1.886	1.852
Co2	2.072	1.982	2.189	1.923	2.340
Co1'	2.077	1.878	1.940	1.917	2.126
